# 侵袭性NK细胞白血病合并CD5^+^CD10⁻单克隆B淋巴细胞增多症及噬血细胞性淋巴组织细胞增生症1例报告并文献复习

**DOI:** 10.3760/cma.j.cn121090-20250920-00429

**Published:** 2026-04

**Authors:** 梅 刘, 小军 刘, 艳 周, 曼娜 张, 迪 郝, 敏 史

**Affiliations:** 1 河北医科大学第二医院检验科，石家庄 050000 Department of Clinical Laboratory, The Second Hospital of Hebei Medical University, Shijiazhuang 050000, China; 2 河北医科大学第二医院血液科，石家庄 050000 Department of Hematology, The Second Hospital of Hebei Medical University, Shijiazhuang 050000, China

## Abstract

侵袭性NK细胞白血病（ANKL）是一种以NK细胞异常增生为特征的罕见血液系统恶性肿瘤，其病情进展迅速且致死率高。本文报道1例罕见的同时合并CD5^+^CD10⁻单克隆B淋巴细胞增多症及噬血细胞性淋巴组织细胞增生症（HLH）的ANKL患者诊治过程并进行相关文献复习。患者为73岁男性，无明显诱因发热伴多部位淋巴结肿大持续1个多月。实验室检查骨髓细胞形态学及骨髓活检发现两群形态不同的异常细胞，经流式细胞术、免疫组化及基因检测符合ANKL合并CD5^+^CD10⁻单克隆B淋巴细胞增多症特征，同时继发HLH。早期给予依托泊苷联合地塞米松治疗，患者在接受培门冬酶为基础联合方案化疗3个疗程后，病情恶化，因多器官功能衰竭抢救治疗无效死亡。

## Abstract (en)

Aggressive natural killer（NK）-cell leukemia（ANKL）is a rare hematological malignancy characterized by the abnormal proliferation of NK cells. It progresses rapidly and has a high mortality rate. This article reports the diagnosis and treatment of a rare case of a patient with ANKL and concurrent CD5^+^CD10⁻ monoclonal B-cell lymphocytosis and hemophagocytic lymphohistiocytosis (HLH), and the relevant literature was reviewed. The patient was a 73-year-old male who presented with fever and lymphadenopathy in multiple areas without any obvious cause for over 1 month. Laboratory examinations, including bone marrow cell morphology and biopsy, revealed two distinct populations of abnormal cells. Flow cytometry, immunohistochemical staining, and gene testing confirmed the diagnoses of ANKL and CD5^+^CD10⁻ monoclonal B-cell lymphocytosis, with concurrent HLH. The patient was initially treated with etoposide and dexamethasone. After receiving three courses of chemotherapy based on pegaspargase and other combination regimens, the patient's condition deteriorated. Resuscitation treatment was ineffective; the patient died due to multiple organ failure.

侵袭性NK细胞白血病（ANKL）是一种罕见的与EB病毒（EBV）密切相关的恶性淋巴增殖性疾病，发病率约占淋巴造血系统肿瘤的0.1％[1]，中位发病年龄约42岁，突出特点为外周血或骨髓中NK细胞来源的大颗粒淋巴细胞增多，临床具有高侵袭性、进展快，易合并或继发噬血细胞性淋巴组织细胞增生症（HLH），预后极差[2]–[3]。ANKL同时合并其他类型淋巴细胞克隆性疾病罕见。近期我院收治1例同时合并HLH及CD5^+^CD10⁻单克隆性B淋巴细胞增多症的ANKL老年患者，其临床和实验室特征较为复杂，诊断过程曲折，现将诊疗经过报道如下，并进行文献复习。

## 病例资料

患者，男，73岁，1个多月前因无明显诱因发热伴纳差，就诊于当地医院，腹部CT示腹膜后多发肿大淋巴结，EBV阳性，为求进一步诊治，2024年6月11日就诊于我院血液科。查体：颈部及腋窝可触及淋巴结，胸骨无压痛，双肺呼吸音粗，肝脾肋下未触及。血常规：WBC 7.87×10^9^/L、HGB 82 g/L、PLT 72×10^9^/L。血清铁蛋白升高，纤维蛋白原降低。血清EBV核酸检测DNA：9.34×10^6^拷贝数/ml。EBV感染淋巴细胞亚群检测EBV-DNA载量，B细胞：1.40×10^4^拷贝数/ml；CD4^+^T细胞：6.50×10^4^拷贝数/ml；CD8^+^T细胞：4.70×10^4^拷贝数/ml；NK细胞：1.40×10^7^拷贝数/ml。NK细胞活性14.4％（提示NK细胞活性减低）。可溶性CD25/白细胞介素2受体（sCD25/IL-2Ra1）6 348.59 pg/ml。浅表淋巴结超声检查：双侧腹股沟区可探及数个低回声包块，最大者位于左侧约3.8 cm×1.4 cm，双侧腋窝、左侧锁骨上窝、颈部双侧均可探及多个淋巴结。PET-CT检查：横膈上下、双侧腋窝、肝门区、双侧腹股沟，均可见多发大小不等淋巴结，最大者位于左侧髂外血管走行区，约2.4 cm×1.1 cm，伴^18^F-氟代脱氧葡萄糖（FDG）摄取异常增高，最大标准摄取值（SUVmax）为2.8；肝体积增大，肋下约6.4 cm，伴^18^F-FDG摄取异常增高，SUVmax为4.0；脾体积增大，形态欠规整，伴^18^F-FDG摄取异常增高，SUVmax为6.6。

细胞形态学检查结果显示外周血涂片大颗粒淋巴细胞占8％（图1A）。骨髓涂片显示有核细胞增生活跃，噬血细胞占1.5％（图1B），发现2种不同形态的异常淋巴细胞（占21.0％），其中一部分细胞体积较大、核形不规则、染色质粗糙，可见核仁，胞质中等丰富，染灰蓝色，可见大小不一嗜天青颗粒，疑似大颗粒淋巴细胞（图1C）；另一部分细胞体积小，核呈圆形或不规则形，染色质较细致，核仁模糊，胞质中等丰富，染深蓝色，无明显颗粒，可见绒毛状突起、空泡及拖尾（图1D）。后续骨髓活检发现两群形态不同的异常细胞浸润，一群细胞灶状分布，核类圆形或略不规则、未见明显核仁；另一群细胞弥漫分布，体积中等、核不规则、核仁不明显；免疫组化显示：CD23（灶状+）、CD20（灶状+）、PAX-5（灶状+）、CD3（部分+）、CD5（灶状+）、CD4（−）、CD8（−）、CD56（较多+）、CD10（−）、CyclinD1（−）、SOX-11（−）、LEF-1（灶状+）、TIA-1（部分+）、GranB（部分+）、Ki-67（部分+），结果提示骨髓内可见两种异常细胞病灶，一种具有NK细胞表型病灶；一种具有单克隆B淋巴细胞病灶。流式细胞术（FCM）检测结果显示两群异常细胞：一群异常细胞占有核细胞24.90％，免疫表型为mCD3⁻、cCD3^+^、CD2^+^、CD7^+^、CD5⁻、CD56^+^、CD16^dim+^、CD11C^dim+^、CD4⁻、CD8⁻、CD94^+^、CD45RA⁻、CD45RO⁻、TDT⁻、MPO⁻、CD1a⁻、CD34⁻、Perforin^+^、GranzymeB^+^，同时KIR检测示CD158各亚群阳性率均极低，为克隆性NK细胞特征；另一群为单克隆B淋巴细胞占11.69％，免疫表型为CD19^+^、CD200^+^、CD38⁻、cKappa⁻、FMC7⁻、CD79b⁻、CD10⁻、CD103⁻、sIgD⁻，弱表达CD20、CD22、CD23、CD5、cLambda、sIgM。染色体核型分析：46,XY[20]。基因检测：IGH、IGK、IGL基因克隆性重排阳性；TCRβ克隆重排阳性，TCRγ、TCRδ均阴性。

**图1:**
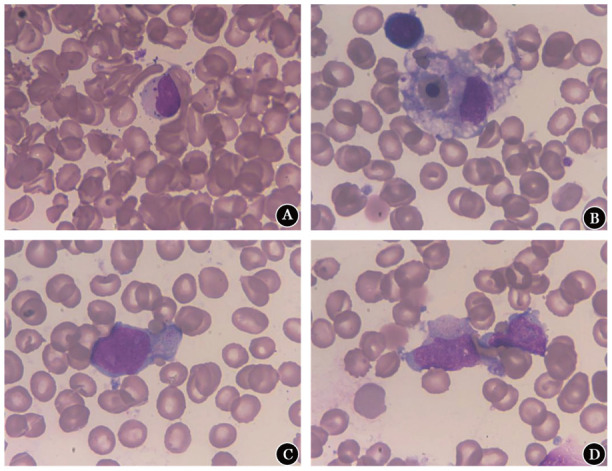
侵袭性NK细胞白血病患者外周血及骨髓涂片细胞形态 **A** 血涂片，颗粒淋巴细胞（瑞-姬染色，×1 000）；**B** 骨髓涂片，噬血细胞（瑞-姬染色，×1 000）；**C** 骨髓涂片，颗粒淋巴细胞（瑞-姬染色，×1 000）；**D** 骨髓涂片，带有伪足及拖尾淋巴细胞（瑞-姬染色，×1 000）

综合以上实验室检查结果，临床最终诊断为ANKL合并CD5^+^CD10⁻单克隆性B淋巴细胞增多症及HLH，2024年6月26日给予VEDP方案（长春新碱+依托泊苷+地塞米松+培门冬酶）诱导化疗3个疗程，患者病情明显好转，发热、水肿症状消失。复查骨髓细胞形态学未见明显异常，FCM微小残留病（MRD）示单克隆B淋巴细胞占5.81％，mCD3⁻CD56^+^ NK细胞占有核细胞0.70％，KIR检测未见明显异常，患者病情平稳，遂出院。2024年9月10日患者第4次入院前10 d无明显诱因出现发热，血常规：WBC 26.60×10^9^/L、HGB 85 g/L、PLT 35×10^9^/L，超敏C反应蛋白及N端-B型钠尿肽前体明显升高，CT胸部平扫示两肺间质性病变较前加重，积极给予经验性抗感染治疗同时给予激素对症处理，并拟行相关检查，但患者病情进展迅速，2024年9月11日患者出现寒战、血氧饱和度下降，经积极抢救仍未能改善状况，患者最终意识丧失，因呼吸循环衰竭死亡。

## 讨论及文献复习

第五版世界卫生组织（WHO）造血与淋巴组织肿瘤分类标准中ANKL属于成熟T/NK细胞肿瘤范畴[4]。ANKL患者通常B症状（不明原因发热、盗汗及体重减轻）明显；肝、脾、淋巴结肿大；80％以上患者EBV阳性[4]；大多数ANKL患者肿瘤细胞可侵犯外周血、骨髓和组织，肿瘤细胞形态学特点为核形不规则，染色质较粗，部分细胞可见核仁1～3个，胞质中等丰富，可见粗大紫黑色嗜碱性颗粒，部分细胞可见拖尾及空泡[4]–[6]。本例患者为73岁老年人，临床表现为发热，肝、脾以及多部位肿大淋巴结，肝功能损伤，EBV核酸定量检测病毒载量明显升高且EBV-DNA（NK细胞）拷贝数增加，血常规结果显示两系血细胞减少，外周血和骨髓细胞形态学均观察到大颗粒淋巴细胞，符合ANKL的临床表现和细胞形态特点。

本例患者细胞形态学显示的另一群异常细胞病灶，经FCM、免疫组化检测证实为CD5^+^CD10⁻单克隆B淋巴细胞。同时本例患者脾大，骨髓中噬血细胞易见，血清铁蛋白水平升高，低纤维蛋白原血症，NK细胞活性减低，sCD25/IL-2Ra1≥6 400 pg/ml，符合《中国噬血细胞综合征诊断与治疗指南（2022年版）》[7]对于HLH的诊断标准。因此该患者经骨髓细胞形态学、免疫分型、细胞遗传学和分子生物学（MICM）综合分析最终诊断为ANKL合并CD5^+^CD10⁻单克隆B淋巴细胞增多症及HLH。既往文献[8]曾报道1例ANKL合并EBV阳性T细胞淋巴瘤，但ANKL合并单克隆B淋巴细胞增多症及HLH在国内外少有报道。

由于ANKL的临床过程呈侵袭性、爆发性进展。Li等[9]对47例ANKL患者进行回顾性分析，结果提示，当骨髓中异常NK细胞比例低于5％时，FCM免疫表型在早期诊断ANKL中具有更高的特异性和敏感性。另有学者应用FCM分析骨髓淋巴瘤细胞免疫表型特征对57例淋巴瘤相关HLH的诊断效能，结果证实FCM对T/NK细胞淋巴瘤相关HLH具有重要的诊断价值[10]。根据第五版WHO造血与淋巴组织肿瘤分类标准，NK细胞典型的FCM免疫表型为：CD2^+^、sCD3⁻、cCD3^+^、CD56^+^、CD16^+^、FASL^+^、细胞毒性蛋白如颗粒酶B（GranzymeB）、TIA1、穿孔素均阳性[4]。本例FCM免疫表型符合克隆性NK细胞的特征。而另一群肿瘤细胞CD19^+^，CD200^+^，CD10⁻，弱表达CD5、CD20、CD23，前向散射光（FSC）及侧向散射光（SSC）偏小，其免疫表型特点与CD5^+^的慢性淋巴细胞白血病/小淋巴细胞淋巴瘤（CLL/SLL）及套细胞淋巴瘤（MCL）相近。但本例血常规示淋巴细胞绝对值为3.90×10^9^/L，多处淋巴结肿大，临床特征暂不符合CLL/SLL的诊断。《套细胞淋巴瘤诊断与治疗中国指南（2022年版）》[11]中提示MCL典型的免疫表型为CD5、CD19、CD20阳性，强表达sIgM、CD10。免疫组化CyclinD1核内强阳性是MCL相对特异的免疫标志，经典型MCL常伴有SOX11阳性。本例免疫组化检查CyclinD1和SOX11均阴性，排除了MCL的可能。综上，FCM在B淋巴细胞增殖性疾病的鉴别中仍具有局限性，需要病理组织学及免疫组化检查进一步证实。

HLH既可以作为淋巴瘤的初始表现，又可在淋巴瘤复发、难治或者终末期时出现，淋巴瘤相关HLH（LA-HLH）常继发于T细胞淋巴瘤和NK细胞淋巴瘤，分别占36％和27％[12]。Cheng等[13]对86例LA-HLH患者的临床特点、治疗及预后因素进行回顾性研究，发现T/NK细胞淋巴瘤患者发生HLH的概率高于B细胞淋巴瘤患者，提示T/NK细胞淋巴瘤可能是HLH发生的主要原因。国内牛挺教授团队[14]分析了94例LA-HLH临床特征与预后，发现T/NK细胞LA-HLH较B细胞LA-HLH患者年龄小，同时T/NK细胞LA-HLH患者基线纤维蛋白原低于B细胞LA-HLH患者，可能与T/NK细胞淋巴瘤患者中T细胞过度活化，产生γ干扰素上调巨噬细胞表面组织因子表达，进而促进异常凝血、消耗纤维蛋白相关[15]。

ANKL的分子发病机制主要与JAK/STAT和RAS/MAPK信号通路相关，涉及表观遗传修饰因子和免疫检查点分子的基因突变[4],[6]。Huang等[16]综合基因组学分析29例ANKL患者外周血样本，发现48％的ANKL患者JAK/STAT通路突变，而细胞外STAT3刺激IL-10水平明显升高，并提出JAK/STAT-MYC生物合成轴可能成为ANKL新的治疗靶点。本例未进行分子生物学检测。

目前ANKL继发HLH尚无标准统一的治疗方案，成人ANKL患者化疗多采用以左旋门冬酰胺或培门冬酶为基础的联合化疗方案如SMILE、GDPE-L、GEMOX-L、EPOCH-L方案[17]。Jung等[18]报道在未行allo-HSCT的情况下，ANKL患者即使对诱导化疗有反应，也仅有10％～20％存活超过1年。Shen等[19]分析71例ANKL患者的总生存期，提出将抗PD-1抑制剂整合到化疗方案中能够显著提升ANKL患者的生存率。随着新一代测序技术发展，Dufva等[20]对14例ANKL患者进行全外显子测序，揭示了JAK-STAT通路在NK细胞恶性肿瘤发病机制中的作用，提出JAK和BCL2抑制剂对NK细胞有高度敏感性。Fujimoto等[21]分析了59例ANKL患者接受allo-HSCT的治疗结局，患者总体1年和5年总生存率分别为33.9％和27.3％，表明allo-HSCT可延长部分ANKL患者生存期，但未来仍需大量病例数据研究和优化治疗策略。本例采用以培门冬酶为基础的联合化疗方案，早期出现HLH给予依托泊苷联合地塞米松治疗，然而本例初诊存在骨髓浸润，继发HLH预后极差，生存期仅76 d。

综上，ANKL在临床中罕见且起病急骤。国内外对其临床研究较少，既往文献多聚焦于ANKL继发HLH的个案报道，而本病例却罕见地同时叠加单克隆B淋巴细胞增多症及HLH，临床症状复杂且缺乏特异性克隆性标志和分子遗传学特征。本病例诊断的关键在于检验医师通过骨髓涂片镜检第一时间同时观察到NK细胞与单克隆B淋巴细胞并与临床直接沟通，通过MICM多维度综合分析从而精准诊断，为罕见复合型血液肿瘤早期制定个性化诊疗方案提供可借鉴路径。
